# 诱导痰、胸水和纤维支气管镜活组织端粒酶活性的联合检测对肺癌的诊断价值

**DOI:** 10.3779/j.issn.1009-3419.2010.02.09

**Published:** 2010-02-20

**Authors:** 红梅 李, 峰 滑, 诚 赵, 光振 刘, 清华 周

**Affiliations:** 1 300052 天津，天津医科大学总医院，天津市肺癌研究所，天津市肺癌转移与肿瘤微环境重点实验室 Tianjin Key Laboratory of Lung Cancer Metastasis and Tumor Microenvironment, Tianjin Lung Cancer Institute, Tianjin Medical University General Hospital, Tianjin 300052, China; 2 266003 青岛，青岛大学医学院附属医院肿瘤中心 Cancer Center, the Affiliated Hospital of Medical College, Qingdao University, Qingdao 266003, China

**Keywords:** 端粒酶, 胸腔积液, 肺肿瘤, 诊断, Telomerase, Pleural effusion, Lung neoplasms, Diagnosis

## Abstract

**背景与目的:**

已有的研究表明, 端粒酶的异常活化与肺癌的发生、发展及转归密切相关, 端粒酶是目前已知广谱的肿瘤分子标记物, 是诊断和治疗肺癌的重要分子标志物之一。本研究旨在探讨诱导痰、胸水和纤维支气管镜活检组织中端粒酶活性的联合检测对肺癌的诊断价值。

**方法:**

对80例肺癌伴胸水患者和50例肺部良性病变伴胸水患者, 应用TRAP-PCR-ELISA方法分别检测其诱导痰、胸水和纤维支气管镜活检组织中端粒酶的活性。

**结果:**

肺癌伴胸水患者诱导痰、胸水和纤维支气管镜活组织中端粒酶的活性均明显高于肺部良性病变伴胸水患者(*P* < 0.001), 不同病理类型的肺癌患者间三种标本的端粒酶活性差异无统计学意义(*P* > 0.05)。诱导痰、胸水和纤维支气管镜活组织中端粒酶活性的检测对肺癌的诊断敏感性分别为62.5%(50/80)、46.3%(37/80)和60.0%(48/80);特异性分别为72.0%(36/50)、66.0%(33/50)和70.0%(35/50);准确性分别为66.2%(86/130)、53.8%(70/130)和63.8%(83/130);三项联合检测的敏感性、特异性和准确性分别为85.0%(68/80)、78.0%(39/50)和82.3%(107/130), 其中敏感性与单独诱导痰、胸水和纤维支气管镜活组织的敏感性差异有统计学意义(*P* < 0.01)。

**结论:**

诱导痰、胸水和纤维支气管镜活组织端粒酶活性的联合检测具有更高的敏感性, 可以提高肺癌的诊断率。

## Abstract (en)

**Background and objective:**

It has been proven that telomerase activation correlates with the carcinogenesis, aggressiveness and turnover of lung cancer.Telomerase is one of the improtant molecular biomarkers for diagnosis and targeting therapy in lung cancer.The aim of this study is to investigate the diagnostic value of the combined determination of telomerase activity in induced sputum, pleural effusion and fiberobronchoscopic biopsy in lung cancer patients.

**Methods:**

The technique of TRAP (telomeric repeat amplification protocal)-PCR-ELISA was employed to detect telomease levels of induced sputum, pleural effusion and fiberobronchoscopic biopsy in 80 lung cancer patients with pleural effusion and 50 benign pulmonary disease patients with pleural effusion.

**Results:**

Telomemse levels of induced sputum, pleural effusion and fiberobronchoscopic biopsy were all significantly higher in patients with lung cancer than those with benign pulmonary disease (*P* < 0.001).There was no significant difference in the level of telomerase activity between different pathologic types (*P* > 0.05).The sensitivity of induced sputum, pleural effusion and fiberobronchoscopic biopsy were 62.5% (50/80), 46.3% (37/80) and 60.0% (48/80), respectively.The specificity were 72.0% (36/50), 66.0% (33/50) and 70.0% (35/50), respectively.The overall accuracy were 66.2% (86/130), 53.8% (70/130) and 63.8% (83/130), respectively.The sensitivity, specificity and overall accuracy of combined induced sputum, pleural effusion and fiberobronchoscopic biopsy were 85.0% (68/80), 78.0% (39/50) and 82.3% (107/130), respectively.The sensitivity of telomease level in combined detection for diagnosis of lung cancer was much higher than that in single sample detection (*P* < 0.01).

**Conclusion:**

The sensitivity of telomease activity in combined three samples was the highest.It can further improve the accuracy for the diagnosis of lung cancer with pleural effusion.

肺癌是我国最常见的恶性肿瘤, 发病率及死亡率均位于所有恶性肿瘤首位, 危害极大。早期发现、早期诊断和早期治疗是提高肺癌生存期的关键。大量研究^[1]^表明, 端粒酶的活化是大部分肿瘤发生的前提条件, 端粒酶活性表达存在于肺癌发生的早期阶段, 端粒酶的异常活化与肺癌的发生、发展及转归密切相关。以往文献^[2]^报道的肺癌端粒酶活性检测标本多来源于手术切除标本、纤支镜活检标本或肺泡灌洗液等, 这些方法均存在一定的局限性, 有研究表明肺癌患者痰或恶性胸腔积液中端粒酶的活性高度表达, 在肺癌的早期诊断中具有重要作用。本文采用端粒重复序列扩增-酶联免疫吸附实验(TRAP-PCR-ELISA)的方法分别测定了肺癌伴胸水患者和良性病变伴胸水患者的诱导痰、胸水和纤维支气管镜活组织端粒酶的活性, 探讨端粒酶联合检测对肺癌的诊断价值。

## 1 材料与方法

### 1.1 临床资料

2007年1月-2008年12月确诊的80例肺癌伴胸水患者, 其中男性50例, 女性30例, 年龄49岁-76岁, 平均年龄59.5岁; 鳞癌39例, 腺癌21例, 小细胞癌15例, 大细胞癌5例。肺部良性病变患者50例, 均伴有胸水, 其中男性32例, 女性18例, 年龄47岁-73岁, 平均龄57.5岁; 其中结核性胸膜炎25例, 肺炎18例, 支气管扩张5例, 肺脓肿2例。肺癌组患者经病理学或细胞学证实并且未做过治疗。肺部良性病变组选用呼吸系统常见疾病并排除其它部位的肿瘤的患者。

### 1.2 诱导痰标本的采集与处理

肺癌患者及良性病变组均收集诱导痰。诱导方法^[3]^为:常规吸入沙丁胺醇200 μg, 然后超声雾化吸入流量为1 mL/min的3%-5%的高渗盐溶液20 min, 整个过程鼓励患者咳嗽, 收集痰液3 mL-5 mL于无菌杯中, 立即用4 ℃无菌生理盐水洗涤两遍, 加入等量的1 moL/L NaOH碱液, 置4 ℃冰箱24 h碱化, 取溶解痰液于4 ℃、3 000 r/min, 离心10 min, 弃上清液。沉淀重悬于PBS中, 4 ℃、3 000 r/min, 再次离心10 min, 弃上清液, 沉淀置于-80 ℃冰箱中待用。检测时取出冷冻的标本在冰上解冻后重悬于200 μL的端粒酶裂解液中冰浴、离心后移取上清液至EP管中待用。

### 1.3 胸水细胞提取液的制备

常规行胸腔穿刺术抽取新鲜胸水100 mL送检, 离心(4 ℃, 1 200 r/min, 10 min), 弃上清, 离心细胞置液氮中保存(-196 ℃)。检测前用预冷的PBS缓冲液(pH7.4)漂洗细胞2次, 分别离心(4 ℃, 1 200 r/min, 10 min), 弃上清。加预冷的端粒酶裂解缓冲液, 0 ℃孵化30 min, 离心(4 ℃, 12 000 r/min, 20 min), 吸出上清液(含端粒酶), 取少许测定蛋白浓度, 将细胞提取物稀释至3 μg/μL。

### 1.4 纤维支气管镜活组织检查

采用Olympus CV 2260型电子纤维支气管镜对所有患者进行检查, 采取活检的肺组织, 用PBS液冲洗后用液氮速冻置于-80 ℃冰箱保存。检测时取冰冻活检组织, 剪碎, 加入100 μL细胞裂解液充分混匀, 水浴30 min, 4 ℃、1 600 r/min离心20 min, 收集上清液置于-80 ℃冰箱冻存备用。在操作纤支镜前常规进行心电图、胸片或胸部CT检查、血常规及出、凝血时间检查。纤维支气管镜未能证实的肺癌患者均通过经皮肺穿或开胸活检、痰中或胸水中检测脱落细胞最终得以证实。

### 1.5 端粒酶活性测定

采用TRAP-ELISA法, 将上述各种标本的沉淀细胞(约1×10^5^-1×10^6^)置于1.5 mL离心管, 150 μL裂解液抽提端粒酶。端粒酶活性和聚合酶链反应(PCR)扩增反应系统:5×端粒重复扩增反应混合液10 μL, TaqDNA多聚酶(5 U/μL)0.4 μL, 样品提取液2 μL, 蒸馏水37.6 μL。采用PCR扩增仪, 酶反应:30 ℃, 30 min。PCR循环扩增:94 ℃, 30 s; 55 ℃, 30 s, 35个循环。阴性对照加2 μL裂解液; 阳性对照加1 μL端粒酶质控模板溶液。扩增产物经包被好的微量96孔板捕获后, 加人辣根过氧化物酶标记的抗二硝基苯抗体(Anti-DNP)孵育1 h后洗涤, 四甲基联苯胺(TMB)显色10 min。MK3酶标仪(Dragon Lab System公司)上用双波长法测定各孔波长450 nm吸光度(A_450_)、波长690 nm吸光度(A_690_)的值, A_690_为参考, 用于消除孔间误差。端粒酶活性为△A= A_450_-A_690_。△A大于阴性对照孔的2.0倍即判定为端粒酶阳性。敏感性=肺癌组测定指标的阳性例数/肺癌组总例数; 特异性=良性疾病组测定指标的阴性例数/良性疾病组的总例数; 准确性=(真阳性数+真阴性数)/研究总例数。

### 1.6 统计学方法

采用SPSS 12.0统计软件包, 端粒酶活性定量值以Mean±SD表示, 比较采用*t*检验; 不同病理类型的肺癌伴胸水患者间诱导痰、胸水和纤维支气管镜活组织端粒酶活性的比较采用方差分析; 端粒酶活性阳性率比较用两样本率的*χ*^2^检验。以*P* < 0.05为差异有统计学意义。

## 2 结果

80例肺癌患者通过纤维支气管镜检查确诊为肺癌的有60例, 胸水中检出肿瘤细胞的12例, 经皮肺穿活检或淋巴结活检的有8例。肺癌伴胸水患者和肺部良性病变伴胸水患者在诱导痰、胸水和纤维支气管镜活组织中端粒酶活性的定量比较见表 1, 结果显示肺癌伴胸水患者三种标本中端粒酶的活性均明显高于肺部良性病变患者(*P* < 0.001)。

**1:** 肺癌伴胸水和肺部良性病变伴胸水患者诱导痰、胸水和纤维支气管镜活组织端粒酶的活性（Mean±SD） The expression of telomerase activity in induced sputum, pleural effusion and fiberobronchoscopic biopsy in lung cancer patients and benign pulmonary disease patients with pleural effusion (Mean±SD)

Sample	Benign lung disease (*n*=50)	Lung cancer patients (*n*=80)	*t*	*P*
Induced sputum	0.091±0.110	0.488±0.163	15.186	< 0.001
Pleural effusion	0.075±0.009	0.376±0.123	17.252	< 0.001
Fiberobronchoscopic biopsy	0.088±0.108	0.463±0.144	15.833	< 0.001

不同病理类型的肺癌患者三种标本中端粒酶活性的比较见表 2(经方差齐性检验四组不同病理类型的肺癌患者的总体方差相同), 其三种标本的端粒酶活性差异无统计学意义(*P* > 0.05)。

**2:** 不同病理类型肺癌伴胸水患者诱导痰、胸水和纤维支气管镜活组织端粒酶的活性（Mean±SD） The expression of telomerase activity in induced sputum, pleural effusion and fiberobronchoscopic biopsy in lung cancer patients with different pathological types (Mean±SD)

Sample	Squamous (*n*=39)	Adenocarcinoma (*n*=21)	Small cell (*n*=15)	Big cell (*n*=5)	*F*	*P*
Induced sputum	0.469±0.153	0.480±0.163	0.478±0.159	0.458±0.160	1.67	> 0.05
Pleural effusions	0.374±0.120	0.356±0.112	0.379±0.131	0.369±0.123	1.71	> 0.05
Fiberbronchoscopic biopsy	0.493±0.144	0.464±0.138	0.463±0.132	0.483±0.140	1.74	> 0.05

三种标本中端粒酶活性的检测对肺癌的诊断敏感性、特异性和准确性见表 3, 其中三种标本联合检测敏感性的结果判断标准为:三种标本有一个以上检测为阳性即判断为阳性, 三种标本全为阴性即判断为阴性。联合检测的敏感性与单独检测相比差异有统计学意义(*P* < 0.01)。

**3:** 诱导痰、胸水和纤维支气管镜活组织端粒酶活性的诊断价值 The diagnotic value of telomerase activity in induced sputum, pleural effusion and fiberobronchoscopic biopsy

Diagnotic value	Specificity (%)	Overall accuracy (%)	Sensitivity (%)	*χ*^2^	*P*
Combination	78.0 (39/50)	82.3(107/130)	85.0 (68/80)		
Induced sputum	72.0 (36/50)	66.2 (86/130)	62.5 (50/80)	10.461	< 0.01
Pleural effusions	66.0 (33/50)	53.8 (70/130)	46.3 (37/80)	26.625	< 0.001
Fiberbronchoscopic biopsy	70.0 (35/50)	63.8 (83/130)	60.0 (48/80)	12.539	< 0.001

## 3 讨论

端粒是真核生物染色体末端包含着独特重复片段的特殊结构, 在保护染色体和染色体复制方面起着重要作用。线性染色体末端的DNA合成是不完全的, 随着每次细胞分裂, 端粒片段会进行性缩短, 最终会导致衰老细胞的凋亡或程序性死亡。端粒长度被认为是细胞的“有丝分裂钟”, 而端粒酶作为一种特殊的逆转录酶, 能够以自身RNA为模板, 合成端粒末端重复(TTAGGG)n, 对稳定端粒长度从而使细胞获得无限增殖能力和不致死性, 成为永生细胞, 甚至恶性转变, 发挥重要作用^[4]^。端粒酶是迄今为止发现的较为广谱的肿瘤标志物^[5]^, 已成为最有希望的抗肿瘤的靶点之一^[6]^。端粒酶在肺癌组织中选择性的高表达, 为以端粒酶为靶点进行肺癌的基因治疗提供了分子生物学基础^[7]^。90%以上癌细胞表达端粒酶活性, 虽然其端粒长度很短, 但由于端粒酶的激活维持了端粒长度, 因而有可能获得无限增殖, 而端粒酶活性增高不存在于正常组织和体细胞^[8]^。

Sen等^[9]^分别检测了42例可疑肺癌患者及10例良性肺病变患者的痰液、支气管肺泡灌洗液(bronchoalveolar lavage, BAL)和活检(气管镜)标本的端粒酶活性, 结果表明, 42例可疑肺癌患者中痰液、BAL、活检标本端粒酶阳性率分别为81.6%、68.4%、86.8%, 10例良性肺病变者仅1例BAL阳性低表达, 随后被证实有大量的炎性细胞。评价分析表明端粒酶鉴别良、恶性肺癌的敏感性和特异性, 在痰标本分别为81.6%、100%, 在BAL标本为68.4%、100%, 而活检标本为86.8%、100%。近年来, 国内外不少学者^[10-12]^通过对肺癌患者痰脱落细胞、纤维支气管镜刷检物及胸水脱落细胞端粒酶活性的检测, 证明端粒酶在上述组织中高表达, 说明端粒酶在肺癌的生成和发展方面具有重要作用。端粒酶活性的表达与恶性肿瘤的侵袭转移及恶性转化有关^[13, 14]^, 随着肿瘤分化程度由高到低, 端粒酶阳性率呈升高趋势。

本研究对80例肺癌伴胸水患者和50例肺部良性病变伴胸水患者, 分别检测其诱导痰、胸水和纤维支气管镜活检组织中端粒酶的活性, 结果发现肺癌伴胸水患者诱导痰、胸水和纤维支气管镜活组织中端粒酶的活性分别为0.488±0.163、0.376±0.123和0.463±0.144;肺部良性病变伴胸水患者诱导痰、胸水和纤维支气管镜活组织中端粒酶的活性分别为0.091±0.110、0.075±0.009和0.088±0.108, 肺癌组均明显高于肺部良性病变组。鳞癌、腺癌、小细胞癌和大细胞癌的患者间三种标本的端粒酶活性差异无统计学意义, 表明端粒酶活性检测可作为肺癌筛选的标志物。

本研究还发现, 肺癌伴胸水患者诱导痰、胸水和纤维支气管镜活组织中端粒酶活性的阳性率分别为62.5%(50/80)、46.3%(37/80)和60.0%(48/80), 亦即诱导痰、胸水和纤维支气管镜活组织端粒酶的活性对肺癌的诊断敏感性分别为62.5%、46.3%和60.0%。准确性分别为66.2%、53.8%和63.8%, 诱导痰检测敏感性与纤维支气管镜活组织检测敏感性差异无统计学意义, 胸水检测敏感性较前两者低。三项联合检测的敏感性和准确性分别为85.0%和82.3%, 其中敏感性与单独的诱导痰、胸水和纤维支气管镜活组织的敏感性差异有统计学意义。

有文献^[15]^报道诱导痰中端粒酶活性的检测有助于肺部良恶性疾病的诊断和鉴别诊断。诊断敏感性约为80%;胸水中端粒酶活性的检测对肺癌诊断的敏感性约为45%。有研究表明在纤维支气管镜活组织中, 端粒酶活性在肺癌患者明显高于肺炎患者和正常人。本研究联合检测诱导痰、胸水和纤维支气管镜活组织端粒酶的活性, 对肺癌的诊断敏感性较单项检测大大提高, 由于痰、胸水和纤支镜活检组织标本容易获得, 联合检测对肺癌伴胸水患者的早期诊断有重要意义, 但因样本量较少, 尚需进一步研究证实。
